# Cost of healthy and culturally acceptable diets in Brazil in 2009 and 2018

**DOI:** 10.11606/s1518-8787.2021055003329

**Published:** 2021-11-12

**Authors:** Eliseu Verly, Dayan Carvalho Ramos Salles de Oliveira, Rosely Sichieri

**Affiliations:** I Universidade do Estado do Rio de Janeiro Instituto de Medicina Social Departamento de Epidemiologia Rio de Janeiro RJ Brasil Universidade do Estado do Rio de Janeiro. Instituto de Medicina Social. Departamento de Epidemiologia. Rio de Janeiro, RJ, Brasil

**Keywords:** Healthy Diet, economy, Food preferences, Basic Food, Costs and Cost Analysis

## Abstract

**OBJECTIVE:**

To estimate the lowest cost of a healthy and culturally acceptable diet and to assess the evolution of its cost in the periods 2008–2009 and 2017–2018.

**METHODS:**

We used data on the individual food consumption and food prices from the *Pesquisas de Orçamentos Familiares* (Household Budget Surveys), in the 2008–2009 and 2017–2018. The sample strata of each period were aggregated, forming 108 new strata with geographic and economic homogeneity. Linear programming models generated diets for each new stratum, considering the constraints in two models: model 1 (≥ 400g of fruits and vegetables); and model 2 (≥ 400g of fruits and vegetables, < 2300mg of sodium, sodium/potassium ratio < 1, ≥ 500mg of calcium). Each food could progressively deviate 5g from the observed consumption averages until the models found a solution in each of the strata. The objective function was to minimize the total cost of the diet.

**RESULTS:**

The average observed and optimized costs were R$4.96, R$4.62 (model 1) and R$5.08 (model 2) in 2008–2009, and R$9.18, R$8.69 and R$9.87 in 2017–2018. Models 1 and 2 resulted in an increase of up to 6% and 11% in 2008–2009, and of up to 25% and 34% in 2017–2018 in the lowest income strata. The main changes observed in the two models include the reduction in the amounts of sweetened beverages, sweets, sauces, ready-to-eat foods, and an increase in fruits and vegetables, flour, and tubers.

**CONCLUSION:**

The adequate amount of fruits and vegetables resulted in an increase in costs to some population strata. When the adequacy of calcium, sodium, and potassium was considered, we observed a more significant increase in cost, especially in 2017–2018.

## INTRODUCTION

Fruit and vegetable consumption is an important marker of healthy eating and is often low in many countries around the world. In Latin American countries, including Brazil, estimations suggest that approximately 5% of deaths are attributed to low consumption of fruits and vegetables^1^. Consumption is lower in lower-income groups^2^, which implies that the adoption of adequate eating habits is determined by the socioeconomic conditions of the families. The economic dimension is particularly important in low- and middle-income countries, where a considerable percentage of total income is spent on food. According to the 2017–2018 *Pesquisa de Orçamentos Familiares* (Family Budget Survey), approximately 25% of Brazilian households live on up to two minimum wages, where the percentage spent on food is 22%, although this expenditure was 14% in the general population^3^.

Unlike other countries, *in natura* foods in Brazil are generally cheaper than processed and ultra-processed foods (UPF). In 2009, the estimated average cost of UPF in Brazil was R$2.40/1,000kcal and for *in natura* (fresh) or minimally processed foods was R$1.56/1,000kcal. However, the average price of fruits and vegetables – items that should have their consumption increased – was R$ 4.17/1,000 kcal and R$ 10.36/1,000 kcal^4^. Consequently, the increase in the participation of fruits and vegetables in the diet leads to a potential increase in the total cost of the diet. However, the cost impact will be smaller if fruits and vegetables replace foods with low nutritional value and higher prices. However, this substitution depends on the local culture, specifically on how much people tolerate changes in their usual eating pattern. There is a well-described relationship in national^5^ and international studies^6^ on the impact of tolerance to changes in consumption on the cost of healthy eating. Overall, diet becomes cheaper as more changes to current diet are tolerated. Therefore, estimations incompatible with local realities could generate incompatible values. Especially in countries with a large territorial extension, such as Brazil, food prices and social variations are important.

We have no information on how the increase in the consumption of fruits and vegetables, as well as dietary changes to prevent chronic non-communicable diseases, could impact the cost of food and how this would occur in different economic strata. Linear programming optimizes variables, subject to restrictions expressed as minimum or maximum values that must be achieved. It is also useful in assessing the feasibility of complex problems involving multiple variables and constraints, for example, lower cost and higher nutritional quality. In this context, the results of diet optimization studies indicate the modifications that could be more effective and efficient, that is, higher quality, acceptability, and lower cost^7^. We intend to estimate the lowest cost of healthy and culturally acceptable diets for the Brazilian population and estratifiede by considering income strata between 2008–2009 and 2017–2018.

## METHODS

### Data Source

We used the *Inquéritos Nacionais de Alimentação* (INA - National Dietary Surveys) data, conducted in 2008–2009 and 2017–2018. Both surveys are subsamples of the *Pesquisas de Orçamentos Familiares* (POF - Household Budget Surveys), conducted by the Brazilian Institute of Geography and Statistics (IBGE). Details about the sampling plan are obtained elsewhere^3 , 8^. The POF samples included 55,970 households in 2008–2009 and 57,920 in 2017–2018. INA data mention 34,003 individuals interviewed in 13,569 households, in 2008–2009, and 46,164 individuals in 20,112 households, in 2017–2018. Household data collection in each stratum was evenly distributed over the 12 months to take into account seasonal variations in food consumption and prices.

### Unit of analysis

The optimized diet should be as close as possible to the usual diet observed in the population. However, the individual food consumption estimated for two days of the collection is inaccurate to describe the usual consumption. Thus, we obtained cost estimations for groups of people, representing the average daily cost in the group. We estimated the costs for different sample strata, considering the heterogeneity in food consumption and food prices between the macro and micro-regions of Brazil. The POF sample strata (550 in 2008–2009 and 575 in 2017–2018) were collapsed according to geographic location (26 Brazilian states and the Federal District) and four strata of income in minimum wages *per capita* : ≤ 0.5 minimum wage (MW); > 0.5 and ≤ 1.5 MW; > 1.5 and ≤ 3 MW; and > 3 MW. (The MW in January 2009 was R$415.00; in January 2018, it was R$954.00). This rearrangement totaled 108 new strata in each period (27×4 = 108), referred to henceforth as “strata”, for which the average costs of current and optimized feeding will be estimated.

### Optimization Models Variables

#### Food intake

In 2008–2009, consumption data were obtained from food records. In turn, the 2017–2018 data were obtained through a 24-hour recall, aided by a software specifically developed for the data collection, having the interviews guided by the automated multiple-pass method^9^. Food consumption averages were obtained for all individuals ≥ 10 years of age. The reported foods, totaling 1,103 in 2008–2009 and 1,593 in 2017–2018, were further classified into 123 foods or food groups. This grouping considered variations of the same food, for example, all types of banana were classified as banana, different ways of preparing fish were classified as fish. Foods belonging to the same group, for example, all beef cuts were classified as beef. Alcoholic beverages, coffee, and tea were not considered. The food consumption averages in each stratum were the starting point to obtain the consumption averages optimized in the linear programming models.

We used the 7.0 Version of the *Tabela Brasileira de Composição de Alimentos* (TBCA - Brazilian Table of Food Composition), compiled by the *Centro de Pesquisa em Alimentos* (FoRC - Food Research Center)^10^ of the Universidade de São Paulo to obtain the nutrient content in observed and optimized diets from both surveys. The nutritional composition of each food already grouped corresponded to the average of the food subtypes weighted by their reporting frequencies in each survey. Given the reporting frequencies varied between strata, we generated tables for each stratum of each survey.

#### Food prices

We extracted food prices from the POF collective expenditure booklet databases, considering the quantity and price records of each food purchased over the course of a week. We converted prices per 100g of edible portion by applying cooking and correction factors. For each of the 123 foods or food groups, a corresponding food in the expense booklet was identified in the respective period. The grouped food price corresponded to the average of food prices weighted by their reporting frequencies within each stratum and for each survey. For example, we obtained the average food price to “beef” using the average weighted by the reported purchase frequency of all types of beef cuts. Thus, price variation among strata was preserved. As food prices and household income were collected over 12 months, both were adjusted using the official rates of the *Índice Nacional de Preços ao Consumidor* (INPC - National Consumer Price Index) for a single reference period in each period (January 31, 2009 and January 15, 2018) allowing comparison between households.

### Model Constraints

Preference contraints refer to the maximum and minimum limits in which the amounts of each food in the optimized diets can deviate from the amount of consumption observed in the population, preventing the optimized diets from being culturally or socially unacceptable. Each food could progressively deviate every 5g from the average intake observed in each stratum until the model returned a solution. This was done by running *n* models for each stratum, imposing lower ( *li* ) and upper ( *ls* ) limits that consist respectively from [ *m*_*f* , *g*_ – *d* ] and [ *m*_*f* , *g*_ + *d* ] where *m*_*f* , *g*_ is the average amount of food *f* observed in stratum *g. D* = (5, 10, 15, …, *n* ) is the allowable deviation from the reported observed quantity of *f* . The constraints for *f* were, however, censored at the 5th percentile (lower limit) and 95th percentile (upper limit). We estimated these percentiles for each food, from each region of the country. Firstly, we obtained the average intake of f in each stratum. Then, the 5th and 95th percentiles of the average consumption distribution of f from each region were obtained. Percentiles were used as constraints on strata within each region.

Additional constraints were added for food groups. Similarly to what was described for foods, the amounts of each food group in the optimized diets could not be less than the 5th percentile and greater than the 95th percentile of the distribution of average consumption within each region of the country. Food groups for which the acceptability constraints were applied are described below. Considering the low consumption of fruits and vegetables in the population, this group and all the foods that contain it had their maximum quantity restrictions relaxed until the model found a possible mathematical solution.

For nutritional constraints, we used two separate sets in two models. In model 1, the diet must contain at least 400g of fruits and vegetables. In model 2, the diet must be adequate in relation to the recommendations for the prevention of chronic diseases^11^. The optimized diets were isocaloric in relation to the observed diets. Ultra-processed foods, classified under the NOVA classification^12^, were restricted to their observed consumption amounts or less. The set of nutritional constraints used is described in Table 1 .

**Table 1:** Nutritional constraints imposed on optimization models.

	Model 1	Model 2
Energy (Kcal)	= observed	= observed
Carbohydrates (%kcal)		55%–75%
Proteins (%kcal)	-	
Fats (%kcal)	-	15%–30%
Saturated fat (%kcal)	-	< 10%
Polyunsaturated fat (%kcal)	-	6%–10%
Trans fat (%kcal)	-	< 1%
Sodium (mg)	-	≤ 2,300
Sodium / Potassium	-	≤ 0.9
Calcium (mg)	-	≥ 500
Fruits and vegetables.	≥ 400	≥ 400
Ultra-processed food (g)	≤ observed	≤ observed

### Linear Programming Models

The linear programming data optimization models were developed to identify the best solution (combination of foods that will compose the optimized diets) that satisfies the set of nutritional adequacy constraints at the lowest possible cost. The objective function can be described according to the following formula


$$y=\sum_{i=1}^{i=g}\left(Q_{i}^{otm}\cdot {\text{ preço }}_{i}\right)$$

where: *g* is the food amount, Q_*i*_^otm^ is the amount of food *i* in the optimized menu, and preço_*i*_ is the price of the food per kilo.

### Descriptive Analysis

The average quantities in the observed and optimized diets for the following groups were evaluated: beans (beans and legumes), dairy products (whole and skimmed milk, cheese, yogurt, and other dairy products), fruits, vegetables, tubers (potatoes, cassava, and yams); red meat (beef and pork), poultry, fish and seafood, eggs, oilseeds, oils (butter and margarine); breads, cakes and biscuits, white and brown rice, pasta (noodles, lasagna pasta and the like), flour (cassava flour and *farofa* ), sauces (salad dressings and pasta sauces), sweetened beverages (soft drinks, industrialized juices, and nectars), ready-to-eat foods (pizza, sandwiches, and snacks) and sweets. The relative difference between the optimized and observed cost [(optimized cost-observed cost)/observed cost × 100] was evaluated according to the income classes for the two periods. To allow a direct comparison between periods, we estimated the observed diet cost in 2008–2009 with food prices of 2017–2018. All analyzes were weighted by sample weights, in which the weight of the stratum corresponded to the sum of the weights of their households. Optimization models were run in SAS software.

## RESULTS

The models only returned a feasible mathematical solution for most strata after making the maximum amounts of fruits and vegetables (individual items and groups) more flexible in up to three times the 95th percentile value of the distribution of average consumption. We maintained restrictions at percentiles 5th and 95th for the other foods and groups.

The estimated average diet cost per person/day in Brazil in 2008–2009 was R$4.96, ranging from R$3.80 to R$6.13 in the lower and higher-income strata. In 2017–2018 it was R$9.18, ranging from R$7.53 to R$11.46 in the lower and higher-income strata. The average optimized cost – which only considers changes in the quantities of fruits and vegetables (model 1) – was R$4.62 in 2008–2009, ranging from R$3.56 to R$5.56. The cost was R$8.69 in 2017–2018, ranging from R$7.38 to R$10.42. In this model, the optimized diet cost an average of 6.85% less than the diet observed in 2008–2009, and 5.34% less than the diet observed in 2017–2018. When nutritional restrictions for the prevention of chronic diseases were included (model 2), the average cost increased to R$5.08 in 2008–2009, ranging from R$4.05 to R$6.09, and R$9.87 in 2017–2018, ranging from R$8.65 to R$11.64. In this model, the optimized diet cost was, on average, 2.42% more than the diet observed in 2008–2009 and 7.52% more than the diet observed in 2017–2018. The largest increases in cost (in percentage) were observed for model 2 in the 2017–2018 period, especially in the strata with *per capita* income up to one minimum wage and in the northeast region. The observed diet cost was lower in both periods in the North and Northeast regions when compared to the South and Center-West regions ( Table 2 ).

**Tabela 2:** Average cost (in Reais) of observed and optimized diets and percentage variation in the cost of the optimized diet according to incomea, region, nutritional restrictions (modelsb) and the survey yearc.

	2008–2009			2017–2018		
	
	Observed^d^	Model 1^e^	Model 2^e^	Observed	Model 1	Model 2
*Per capita* income						
< 0.5	3.80 (0.11)	3.56 (-6.31)	4.05 (+6.58)	7.53 (0.18)	7.38 (-1.96)	8.65 (+14.87)
0.5–1	4.42 (0.07)	4.15 (-6.10)	4.62 (+4.52)	8.31 (0.19)	8.02 (-3.49)	9.35 (+12.52)
1–2	4.91 (0.07)	4.66 (-5.09)	5.04 (+2.64)	9.13 (0.18)	8.67 (-5.04)	9.66 (+5.81)
> 2	6.13 (0.05)	5.56 (-9.29)	6.09 (-0.65)	11.46 (0.23)	10.42 (-9.08)	11.64 (+1.57)
Region						
North	5.28 (0.17)	4.91 (-7.00)	5.35 (+1.32)	8.68 (0.23)	8.10 (-6.68)	8.91 (+2.65)
Northeast	4.37 (0.16)	3.96 (-9.38)	4.59 (+5.03)	8.40 (0.27)	7.82 (-6.90)	9.51 (+13.21)
Southeast	5.18 (0.29)	4.91 (-5.21)	5.20 (+0.38)	9.26 (0.57)	8.88 (-4.10)	9.8 (+5.83)
South	5.22 (0.23)	4.81 (-7.85)	5.41 (+3.64)	10.32 (0.47)	9.62 (-6.78)	11.05 (+7.07)
Midwest	5.09 (0.2)	4.80 (-5.69)	5.27 (+3.53)	9.90 (0.39)	9.52 (-3.84)	10.47 (+5.76)
Brazil	4.96 (0.16)	4.62 (-6.85)	5.08 (+2.42)	9.18 (0.28)	8.69 (-5.34)	9.87 (+7.52)

^a^ Household income *per capita* in minimum wages.

^b^ Model 1: adequacy of fruits and vegetables. Model 2: adequacy of fruits and vegetables, macronutrients and fats, calcium, sodium and potassium. Amount of ultra-processed foods less than or equal to the amount observed for both models.

^d^ Average (standard error).

^e^ Cost in Reais (percentage in relation to the cost observed in the period) calculated as: [(optimized cost - observed cost)/observed cost × 100].

**Figure 1:**
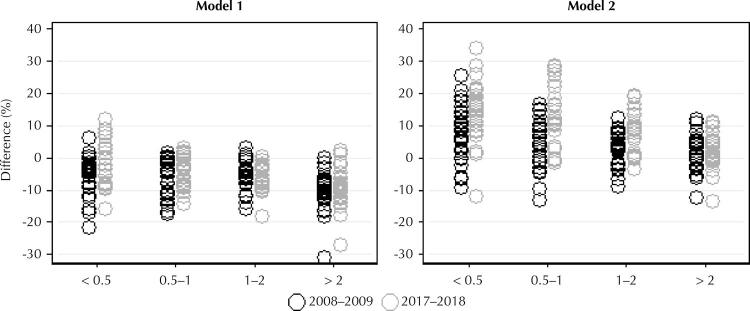
Relative difference (%)a the optimized and observed costs in the strata according to income b, nutritional restrictions (modelsc) e year of survey.

^a^ Calculated [(optimized cost-observed cost)/observed cost] × 100.

^b^ Household income per capita in minimum wages.

^c^ Model 1: adequacy of fruits and vegetables. Model 2: adequacy of fruits and vegetables, macronutrients and fats, calcium, sodium and potassium. Amount of ultra-processed foods less than or equal to the amount observed for both models.

In the lowest income strata, the cost of the optimized diet (model 1) in relation to the observed cost varied from -21% to +6% in 2008–2009 and -15% to +11% in 2017–2018. In the model 2, this variation was from -11% to +25% in 2008–2009 and -11% to +34% in 2017–2018. Also in these income strata, the average cost of the optimized diets in 2008-2009 was higher than the cost of the observed diets in the period in 8% of the strata in the model 1 and in 71% of the strata in the model 2. In 2017–2018 these values were 16% in the model 1 and 82% in the model 2. The increase in the cost related to the optimized diet reached 34% in relation to the cost observed in the strata of up to 1 minimum wage *per capita* in model 2 ( Figure 1 ).

If the 2017–2018 diet were the same practiced in 2008–2009, that is, the 2008–2009 diet with 2017–2018 prices, its cost would be R$9.61. Therefore, it would mean to be R$0.43 more expensive than estimated in 2017–2018.

In the first survey, consumption of rice, dairy products, beans, sweets, fish, and sweetened beverages was higher than in the second survey. In general, the dietary modifications to accommodate the increase in the amounts of fruit and vegetables were similar between the two periods: reduction in dairy products, chicken, sauces, eggs, red meat, *snacks* , and sweetened beverages; and increase in rice and flour. To also accommodate the recommendations for the prevention of NCDs, a more significant increase in fruits and vegetables was needed, totaling 535g in 2008–2009 and 486g in 2017–2018, in addition to an increase in tubers, beans, and dairy products; and a reduction in margarine and butter, past, breads, biscuits, and cake ( Table 3 ).

**Table 3:** Average consumption in grams of foods and food groups in observed and optimized diets according to nutritional constraints (modelsa) and year of surveyb.

	2008–2009			2017–2018		
	
	Observed^c^	Model 1	Model 2	Observed^c^	Model 1	Model 2
Rice	180.80 (5.86)	194.21	180.14	149.54 (4.5)	160.77	148.72
Biscuits	16.54 (0.75)	14.33	9.71	15.49 (0.73)	13.76	10.11
Cakes	12.96 (0.69)	13.40	14.99	11.19 (0.55)	8.84	8.39
Dairy Products	134.35 (5.34)	112.11	147.06	97.65 (4.64)	79.35	117.41
Sweets	21.51 (1.09)	14.48	16.48	14.5 (0.94)	7.43	8.82
Flours	9.18 (1.89)	14.95	15.82	7.83 (1.59)	11.97	12.31
Beans	203.55 (8.14)	203.23	217.03	175.19 (6.47)	179.18	189.79
Poultry	36.15 (1.32)	29.38	35.04	50.57 (1.76)	44.29	53.64
Fruits	177.51 (6.96)	259.75	340.64	166.25 (5.83)	251.64	301.02
Vegetables	60.32 (3.31)	140.25	194.57	66.8 (3.19)	148.36	185.89
Pasta	43.89 (2.13)	46.59	33.51	40.64 (1.69)	43.76	31.76
Sauces	4.46 (0.59)	1.45	1.41	4.52 (0.35)	1.40	2.07
Oilseeds	0.18 (0.03)	0.31	0.34	0.34 (0.06)	0.52	0.58
Oils (butter and margarine)	6.32 (0.19)	8.52	3.52	8.26 (0.31)	8.28	4.79
Eggs	12.35 (0.72)	8.84	13.11	12.74 (0.45)	8.76	12.37
Breads	56.76 (1.49)	58.80	42.21	57.94 (1.44)	60.17	49.53
Fish	28.34 (3.75)	21.49	26.60	18.78 (2.36)	12.47	20.99
Meat (beef and pork)	95.65 (2.43)	78.04	73.81	99.02 (2.27)	82.92	85.83
Ready-to-eat foods	20 (1.58)	14.98	11.54	22.16 (1.25)	15.97	13.94
Sweetened Beverages	123.23 (9.62)	116.53	111.07	80.52 (3.43)	74.17	69.90
Tubers	30.61 (1.73)	27.15	42.64	33.7 (1.79)	29.41	42.65

^a^ Modelo 1: Model 1: adequacy of fruits and vegetables. Model 2: adequacy of fruits and vegetables, macronutrients and fats, calcium, sodium and potassium. Amount of ultra-processed foods less than or equal to the amount observed for both models.

^b^ n = 108 strata for both periods.

^c^ Average (standard error).

The impact of the increase of fruits and vegetables on the amounts of nutrients was more evident for fiber, vitamin A, vitamin C, and folate, which increased by at least 10% in relation to the intake observed for the two periods. On the other hand, there was a reduction in both periods. By including the nutritional constraints in the model 2, we observed a more significant increase for these and other nutrients, such as vitamin E, copper, zinc, and magnesium, in addition to an increase in calcium and potassium and a reduction in sodium, nutrients that had the amounts defined in the model 2 ( Table 4 ).

**Table 4:** Average energy and nutrient intake in observed and optimized diets according to nutritional constraints (modelsa) and year of the surveyb.

	2008–2009			2017–2018		
	
	Observed^c^	Model 1	Model 2	Observed^c^	Model 1	Model 2
Energy (Kcal)	1,696.7 (13.4)	1,696.8	1,696.8	1,699.9 (10.5)	1,699.9	1,699.9
Carbohydrates (g)	2,32.5 (1.65)	252.5	253.6	230.1 (2.0)	251.3	244.2
Protein (g)	77.5 (0.8)	69.8	73.2	78.5 (0.6)	71.2	79.4
Fats (g)	56.3 (0.7)	52.0	50.8	56.8 (0.6)	51.8	51.8
Monounsaturated fat (g)	17.2 (0.2)	15.5	15.3	18.4 (0.3)	17.2	15.8
Polyunsaturated fat (g)	13.4 (0.2)	12.5	12.8	14.2 (0.2)	13.0	13.1
Saturated fat (g)	19.3 (0.3)	17.9	16.8	17.8 (0.2)	15.5	16.3
Trans fat (g)	1.43 (0.03)	1.37	1.09	1.33 (0.02)	1.2	1.07
Fibers (g)	25.1 (0.4)	29.1	32.5	22.7 (0.3)	26.9	29.5
Calcium (mg)	445.1 (12.7)	401.9	508.5	394.1 (12.6)	353.4	500.8
Copper (mg)	1.45 (0.03)	1.55	1.70	1.29 (0.02)	1.40	1.54
Iron (mg)	11.3 (0.1)	11.3	11.1	10.8 (0.1)	10.8	11.0
Phosphorus, mg	1,001.5 (11.1)	920.8	1.011.8	972.6 (6.8)	895.4	1035
Magnesium, mg	258.7 (3.4)	272.5	300.2	239.7 (2.0)	254.6	282.2
Potassium, mg	2,118.3 (28.8)	2,296.7	2,761.3	2,011.0 (19.2)	2,196.6	2,616.9
Sodium (mg)	2,505.6 (29.1)	2,382	2,238.4	2,376.6 (21.2)	2,264.9	2,260.5
Zinc (mg)	10.8 (0.1)	10.1	10.2	10.8 (0.1)	10.2	10.7
Niacin (mg)	13.8 (0.3)	12.3	12.8	15.1 (0.2)	13.4	15.1
Folate (mcg) ^d^	446.5 (6.7)	477.7	491.5	407.4 (5.3)	439.3	464.2
Vitamin A (EAR) ^e^	518.8 (26.4)	603.1	732.2	429.8 (17.7)	475.3	639.5
Vitamin B1 (mg)	0.89 (0.01)	0.89	0.89	0.93 (0.01)	0.91	0.96
Vitamin B12 (mg)	5.3 (0.18)	4.42	4.64	4.16 (0.12)	3.42	4.02
Vitamin B2 (mg)	1.08 (0.02)	1.03	1.06	0.93 (0.01)	0.89	0.96
Vitamin B6 (mg)	0.66 (0.01)	0.7	0.65	0.62 (0.01)	0.66	0.65
Vitamin C (mg)	124.9 (3.7)	243.4	339.5	117.4 (3.5)	233.3	270.3
Vitamin D	2.66 (0.07)	2.31	2.77	2.11 (0.04)	1.8	2.33
Vitamin E (mg)	5.78 (0.07)	6.34	7.09	6.11 (0.08)	6.76	6.81

^a^ Model 1: adequacy of fruits and vegetables. Model 2: adequacy of fruits and vegetables, macronutrients and fats, calcium, sodium and potassium. Amount of ultra-processed foods less than or equal to the amount observed for both models.

^b^ n = 108 strata for both periods.

^c^ Average (standard error).

^d^ In dietary folate equivalents (DFE).

^e^ In retinol activity equivalents.

## DISCUSSION

This study estimated the lowest cost of healthy and culturally acceptable diets for the periods 2008–2009 and 2017–2018 considering variations in food preferences and food prices in the country. The adequacy of fruits and vegetables could be achieved with a reduction of 6.8% and 5.3% in relation to the observed diet cost in 2008–2009 and 2017–2018, respectively. The inclusion of restrictions related to the prevention of NCD resulted in a more significant increase of 7.5% in 2017–2018. These differences in the impact on the cost of adopting a healthy diet between periods can be explained by an uneven price variation between food groups. From the early 2000s onwards, the price of ultra-processed foods has been decreasing in relation to the price o *in natura* (fresh) and minimally processed foods^13^. In addition, consumption averages for some foods that are markers of healthy eating, such as dairy products, fruits, beans, and fish, were slightly higher in 2008–2009. For comparison purposes, the cost of the 2008–2009 diet, if purchased at 2017–2018 prices, would be R$9.61, that is, 4.7% more than the estimated 2017–2018 diet cost, which was R$9.18. Thus, the adequacy of the diet required fewer modifications and, consequently, lower costs, particularly in model 2, in which the nutritional composition of the food was also considered in the optimization.

The reduction in the average cost of eating with an adequate amount of fruits and vegetables present in the model 1 was possible due to the amount reduction in the other foods. The model optimizes food quantities within the smallest possible range to include the defined amount of fruit and vegetables, i.e., the adequacy does not increase the cost as long as other dietary modifications are also made. Notwithstanding the adequacy of fruits and vegetables has not resulted in an increase in the overall and income stratified average diet cost, in several strata the increase in the cost was necessary. This indicates an important variation in the impact of dietary adequacy on cost across the country. Particularly in the last survey and especially in the lower-income groups, the increase in cost was greater than 30%, in relation to the cost observed for some strata. In fact, adapting the amount of fruits and vegetables in the diet of low-income families seems not to be possible without an increase in the diet cost. A similar study using data from the 2008–2009 survey with the objective of reducing the percentage of caloric participation of UPF and adapting as much as possible the amount of fruits and vegetables in 400g/day without increasing the cost of the diet, reached 350g in the strata of up to half minimum wage *per capita* . In higher income strata, this amount reached 700g^14^. In the model 2, the increase occurred in all income strata, except among those with higher incomes in 2008–2009. The immediate implication of these results is clear. For a considerable percentage of families, diet improvement results in greater spending on food.

The minimum cost of adequate diet was obtained considering minimum and maximum limits, called here acceptability constraints. These restrictions delimit how much each food can “deviate” from the amount reported in each of the periods. Given to the fact we have no information on food preferences in the population, the acceptability limits were derived from the distribution of consumption in the population itself. The rationale is that if people have reported these items, it means they are acceptable to the population. However, the limits from distributions (the 5th and 95th percentiles) were arbitrarily defined, which might be considered a limitations in studies involving sociocultural aspects of the diet – although this is a common procedure in similar studies^6 , 15^. In addition to the sociocultural dimension, the definition of a healthy diet also generates an important impact on costs. When adequate vitamins and minerals are included among the constraints of the models, the cost is, on average, 20% higher in relation to the observed expenditure. This occur at all income levels^5^. However, the cost of an adequate diet of micronutrients has reduced as more flexible acceptability constraints have been introduced into the models. The greater the tolerance to consumption modification, the lower the final cost. It is noteworthy that optimized cost was always higher to lower-income families than observed for all imposed acceptability constraints, which did not occur in the other income strata^5^. We should bear in mind that the estimated cost in this study corresponds to the lowest cost related to the smallest deviation from the usual diet in each of the periods. As in other studies, the cost decreases as more drastic changes are tolerated.

The present study used two sets of nutritional constraints. The first one concerns food quantities and is more realistic, because people are more likely to shop by observing the quantities of each food in the basket. But in the model 2 scenario is important to assess how much adherence to other recommendations for the prevention of NCDs would require changes in diet and in the budget itself. This is important information for health professionals and for the elaboration of guides that should consider strategies to reach goals for key nutrient intakes at the lowest cost and with greater acceptability.

Some methodological issues must be considered when interpreting these results. We assume prices of food, such as cakes, sandwiches, sweets, pizzas, snacks, and fried and baked snacks corresponding to the price of food purchased ready for consumption, even though they can be items prepared at home from the purchase of ingredients. In that case, the average price of these items will be overestimated as these preparations are made at home. On the other hand, for preparations based on cereals, legumes, meats, and pasta - for example rice, beans, lasagna, etc. - we assumed the sum of the price of their ingredients, even if they may have been acquired ready-to-eat in markets or restaurants. In this case, the average price may be underestimated, because preparations are expected to cost more when purchased ready-made than prepared at home. Underreporting of consumption – common in food surveys – certainly leads to an underestimation of the observed diet cost^16^. However, both the observed cost and the optimized cost were calculated for the same average amount of calories in each period; therefore, similarly impacted by underreporting. In the absence of underreporting, the observed and expected costs would be higher than ours. However, it is impossible to state the differences would remain in the same proportion as that found in the study. Underreporting can occur differently between foods^17^. If it was higher in fruits and vegetables, the increase in cost would be smaller than that obtained here, due to the increase in the food amount in this group. We also highlight that changes in optimized diets lead not only to economic and cultural changes in food, but also to aspects of convenience. The increase in fruit and vegetable implies more frequent visits to the market and more time spent on preparation and cooking. Variables not considered in this study.

One of the main points of this study was to consider the wide variation in prices and consumption patterns across the country, optimizing the cost of an adequate diet as similar as possible to the diet observed in each stratum and in each period. The assessment of the mean population diet cost omits the extent to which dietary adequacy impacts strata in different regions of Brazil. However, due to the great day-to-day variation in food consumption, it was necessary to group individuals into strata, once the one-day dietary survey provides a good approximation of the mean usual consumption in the group^18^.

In conclusion, the adequacy of fruit and vegetables in diets may not lead to an increase in the average cost both in 2008–2009 and in 2017–2018. However, for a significant percentage of the strata, the adequacy would demand an increase in spending on food, particularly in the 2017–2018 period. When the adequacy of calcium, sodium, and potassium are also introduced to the optimization models, an increase in the cost of the diet was observed in the two periods.
