# Supplementary material for: Cost of healthy and culturally acceptable diets in Brazil in 2009 and 2018
Source: Rev Saude Publica. 2021 Nov 12;55(Suppl 1):7s. doi: 10.11606/s1518-8787.2021055003329 (PMC8627151; doi:10.11606/s1518-8787.2021055003329)
Supplement: Translation

# Translation: Custo de uma alimentação saudável e culturalmente aceitável no Brasil em 2009 e 2018

**Authors:** Eliseu Verly, Dayan Carvalho Ramos Salles de Oliveira, Rosely Sichieri

**Affiliations:** I Brasil Universidade do Estado do Rio de Janeiro. Instituto de Medicina Social. Departamento de Epidemiologia. Rio de Janeiro, RJ, Brasil

**Keywords:** Dieta Saudável, economia, Preferências Alimentares, Alimentação Básica, Custos e Análise de Custo

## Abstract

**OBJETIVO:**

Estimar o menor custo de uma alimentação saudável e culturalmente aceitável e avaliar a evolução de seu custo nos períodos de 2008–2009 e 2017–2018.

**MÉTODOS:**

Foram utilizados dados de consumo individual dos módulos de consumo alimentar das Pesquisas de Orçamentos Familiares de 2008–2009 e 2017–2018. Os preços dos alimentos foram obtidos do módulo de caderneta de despesa das respectivas pesquisas. Os estratos amostrais de cada período foram reagrupados, formando 108 novos estratos com homogeneidade geográfica e econômica. Modelos de programação linear foram elaborados para gerar dietas para cada novo estrato, considerando as restrições do modelo 1 (≥ 400g de frutas e hortaliças) e modelo 2 (≥ 400g de frutas e hortaliças, < 2300mg de sódio, relação sódio/potássio < 1, ≥ 500mg de cálcio). Cada alimento das dietas observadas poderia desviar progressivamente em 5g a partir das médias de consumo observadas até que os modelos encontrassem uma solução em cada um dos estratos. A função objetivo foi de minimizar o custo total da dieta.

**RESULTADOS:**

Os custos médios observados e otimizados foram de R$4,96, R$4,62 (modelo 1) e R$5,08 (modelo 2) em 2008–2009 e de R$9,18, R$8,69 e R$9,87 em 2017 –2018. Nos modelos 1 e 2 ocorreram incrementos de até 6% e 11% em 2008–2009 e de até 25% e 34% em 2017–2018 na menor faixa de renda. As principais modificações observadas nos dois modelos incluem a redução nas quantidades de bebidas adoçadas, doces, molhos, alimentos prontos para consumo e aumento de frutas e hortaliças, farinhas e tubérculos.

**CONCLUSÃO:**

A adequação da quantidade de frutas e hortaliças acarretou aumento no custo para parte da população. Quando a adequação de cálcio, sódio e potássio foram consideradas, ocorreu um aumento mais expressivo no custo, especialmente em 2017–2018.

## INTRODUÇÃO

O consumo de frutas e hortaliças é um marcador importante de alimentação saudável e frequentemente descrito como baixo em muitos países do mundo. Em países da América Latina, incluindo o Brasil, estima-se que aproximadamente 5% das mortes sejam atribuídas ao baixo consumo de frutas e hortaliças^1^. O consumo é menor nos grupos de menor renda^2^, o que implica que a adoção de hábitos alimentares adequados são determinados pelas condições socioeconômicas das famílias. A dimensão econômica é particularmente importante em países de baixa e média renda, onde um percentual considerável da renda total é gasta com alimentação. De acordo com a Pesquisa de Orçamentos Familiares de 2017–2018, aproximadamente 25% dos domicílios brasileiros vivem com até dois salários mínimos, onde o percentual gasto com alimentação chegou a 22% dos rendimentos totais, enquanto que na população em geral esse gasto foi de 14%^3^.

Diferente de outros países, os alimentos *in natura* no Brasil são, em média, mais baratos que os processados e ultraprocessados (AUP). Em 2009, o custo médio estimado dos AUP no Brasil era R$ 2,40/1.000kcal e de alimentos *in natura* ou minimamente processados era R$ 1,56/1.000kcal. No entanto, o preço médio das frutas e hortaliças – itens que devem ter seu consumo aumentado – era R$ 4,17/1.000kcal e R$ 10,36/1.000kcal^4^. Consequentemente, o aumento na participação de frutas e hortaliças na alimentação acarreta potencial aumento no custo total da dieta. Porém, o impacto no custo será menor se as frutas e hortaliças substituírem preferencialmente alimentos de baixo valor nutricional e de maior preço. Contudo, essa substituição depende da cultura local, mais especificamente, do quanto as pessoas toleram de modificação em seu padrão usual de alimentação. Existe uma relação bem descrita em estudos nacionais^5^e internacionais^6^sobre o impacto da tolerância às mudanças no consumo no custo de uma alimentação saudável. De forma geral, a alimentação se torna mais barata à medida que mais modificações na alimentação atual são toleradas. Portanto, estimar os custos de uma alimentação adequada sem considerar as preferências alimentares pode gerar valores que não são compatíveis com as realidades locais. Particularmente em países com grande extensão territorial, como o Brasil, além da grande variedade no consumo, há também uma variação importante no preço dos alimentos.

Não há informações do quanto o aumento no consumo de frutas e hortaliças, bem como modificações na dieta para prevenção de doenças crônicas não transmissíveis podem impactar no custo da alimentação e como isso ocorreria nos diferentes estratos econômicos. A programação linear é um método para otimização de variáveis, sujeita a restrições expressas como valores mínimos ou máximos que devem ser alcançados. Ela também é útil na avaliação da viabilidade de problemas complexos envolvendo múltiplas variáveis e restrições, por exemplo, menor custo e maior qualidade nutricional. Nesse contexto, os resultados de estudos de otimização da dieta indicam as modificações que poderiam ser mais efetivas e eficientes, ou seja, maior qualidade, aceitabilidade e menor custo^7^. Assim, o objetivo deste estudo é estimar o menor custo de uma alimentação saudável e culturalmente aceitável na população brasileira em estratos de renda e sua evolução entre os períodos de 2008–2009 e 2017–2018.

## MÉTODOS

### Fonte de Dados

Foram utilizados os dados dos Inquéritos Nacionais de Alimentação (INA) conduzidos nos anos de 2008–2009 e 2017–2018. Ambas as pesquisas são subamostras das Pesquisas de Orçamentos Familiares (POF) conduzidas pelo Instituto Brasileiro de Geografia e Estatística (IBGE). Detalhes sobre o plano amostral foram obtidos em outras publicações^3 , 8^. As amostras das POF contemplaram 55.970 domicílios em 2008–2009 e 57.920 em 2017–2018. Nos INA foram entrevistados 34.003 indivíduos em 13.569 domicílios em 2008–2009 e 46.164 indivíduos em 20.112 domicílios em 2017–2018. A coleta de dados domiciliares em cada estrato foi distribuída uniformemente ao longo dos 12 meses para contemplar as variações sazonais de consumo e preços dos alimentos.

### Unidade de Análise

A dieta otimizada deverá ser a mais próxima possível da alimentação habitual observada na população. No entanto, o consumo alimentar individual estimado por dois dias de coleta não descreve com precisão o consumo habitual. Assim, as estimativas de custo foram obtidas para grupos de pessoas, representando o custo médio diário no grupo. Em função da heterogeneidade no consumo alimentar e nos preços dos alimentos entre as macro e microrregiões do país, os custos foram estimados para diversos estratos amostrais. Os estratos das amostras das POF (550 em 2008–2009 e 575 em 2017–2018) foram colapsados segundo localização geográfica (26 estados brasileiros e Distrito Federal) e quatro estratos de renda em salários mínimos *per capita* : ≤ 0,5 salário mínimo (SM); > 0,5 e ≤ 1,5 SM; > 1,5 e ≤ 3 SM; e > 3 SM. (O SM em janeiro de 2009 era R$415,00; em janeiro de 2018 era R$954,00). Esse rearranjo totalizou 108 novos estratos em cada período (27×4 = 108), referidos aqui apenas como “estratos”, para os quais serão estimados os custos médios da alimentação atual e otimizada.

### Variáveis dos Modelos de Otimização

#### Consumo alimentar

Em 2008–2009, os dados de consumo foram obtidos por registro alimentar. Por sua vez, os dados de 2017–2018 foram obtidos por recordatório de 24 horas, auxiliado por um software desenvolvido especificamente para coleta e tendo as entrevistas orientadas pelo método automatizado de múltiplas passagens^9^. As médias de consumo dos alimentos foram obtidas para todos os indivíduos ≥ 10 anos de idade. Os alimentos relatados em um total de 1.103 em 2008–2009 e de 1.593 em 2017–2018 foram posteriormente classificados em 123 alimentos ou grupo de alimentos. Esse agrupamento considerou variações de um mesmo alimento, por exemplo, todos os tipos de banana foram classificados como banana, diferentes modos de preparo de peixes classificados como peixe. Os alimentos pertencentes a um mesmo grupo, por exemplo, todos os cortes de carne bovina foram classificados como carne bovina. Não foram consideradas as bebidas alcoólicas, café e chá. As médias de consumo dos alimentos em cada estrato foram utilizadas como pontos de partida para a obtenção das médias de consumo otimizadas nos modelos de programação linear.

A versão 7.0 da Tabela Brasileira de Composição de Alimentos (TBCA), compilada pelo Centro de Pesquisa em Alimentos (Food Research Center, FoRC)^10^da Universidade de São Paulo foi utilizada para obter o teor de nutrientes em dietas observadas e otimizadas de ambos os inquéritos. A composição nutricional de cada alimento já agrupado correspondeu à média dos subtipos dos alimentos ponderada por suas frequências de relato em cada inquérito. Como as frequências de relato variaram entre os estratos, foram geradas tabelas para cada estrato em cada inquérito.

#### Preços dos alimentos

Os preços dos alimentos foram extraídos dos bancos de dados de caderneta de despesas coletivas das POF, onde há o registro da quantidade e preço de cada alimento adquirido ao longo de uma semana. Os preços foram convertidos em preços por 100g de porção comestível aplicando-se fatores de cocção e correção. Para cada um dos 123 alimentos agrupados, foram identificados seus equivalentes nas cadernetas de despesas dos respectivos períodos. O preço do alimento agrupado correspondeu à média dos preços dos alimentos ponderada por suas frequências de relato dentro de cada estrato e para cada inquérito. Por exemplo, o preço médio do alimento “carne bovina” foi obtido por meio da média ponderada pela frequência de relato de compra de todos os tipos de corte de carne bovina. Assim, a variação de preços entre os estratos foi preservada. Como os preços dos alimentos e os rendimentos das famílias foram coletados ao longo de 12 meses, ambos foram ajustados utilizando as taxas oficiais do Índice Nacional de Preços ao Consumidor Amplo (IPCA) para um único período de referência em cada período (31 de janeiro de 2009 e 15 de janeiro de 2018) permitindo a comparação entre os domicílios.

#### Restrições do modelo

As restrições de preferências referem-se aos limites máximos e mínimos nos quais as quantidades de cada alimento nas dietas otimizadas podem desviar em relação à quantidade de consumo observada na população, evitando que dietas otimizadas sejam cultural ou socialmente inaceitáveis. Cada alimento poderia desviar progressivamente a cada 5g a partir da ingestão média observada em cada estrato até que o modelo retornasse uma solução. Isso foi feito executando *n* modelos para cada estrato, impondo limites inferiores ( *li* ) e superiores ( *ls* ) que consistem respectivamente em [ *m*_*f* , *g*_– *d* ] e [ *m*_*f* , *g*_+ *d* ], onde *m*_*f* , *g*_é a quantidade média do alimento *f* observada no estrato *g* , e *d* = (5, 10, 15, …, *n* ) é o desvio permitido da quantidade observada relatada de *f* . As restrições para *f* foram, no entanto, censuradas ao percentil 5 (limite inferior) e percentil 95 (limite superior). Esses percentis foram estimados para cada alimento dentro de cada região do país. Primeiro, foi obtida a média de ingestão de f em cada estrato. Em seguida, foram obtidos os percentis 5 e 95 da distribuição das médias de consumo de f dentro de cada região. Os percentis foram usados como restrições nos estratos dentro de cada região.

Restrições adicionais foram incluídas para grupos de alimentos. De forma similar ao descrito para os alimentos, as quantidades de cada grupo de alimento nas dietas otimizadas não poderiam ser menores que o percentil 5 e maiores que o percentil 95 da distribuição das médias de consumo dentro de cada região do país. Os grupos de alimentos para os quais foram aplicadas as restrições estão descritos abaixo. Considerando o baixo consumo de frutas e hortaliças na população, este grupo e todos os alimentos que o contém tiveram suas restrições máximas de quantidade relaxadas até que o modelo encontrasse solução matemática possível.

Para as restrições nutricionais foram utilizados dois conjuntos separadas em dois modelos. No modelo 1 a dieta deve conter pelo menos 400g de frutas e hortaliças. No modelo 2, em adição ao modelo 1, a dieta deve estar adequada em relação às recomendações para prevenção de doenças crônicas^11^. As dietas otimizadas serão isocalóricas em relação às dietas observadas. Alimentos ultraprocessados, classificados segundo a classificação NOVA^12^, foram restringidos às suas quantidades observadas de consumo ou menos. O conjunto das restrições utilizadas encontra-se descrita na Tabela 1 .

**Tabela 1:** Restrições nutricionais impostas aos modelos de otimização.

	Modelo 1	Modelo 2
Energia (kcal)	= observado	= observado
Carboidratos (%kcal)		55%–75%
Proteínas (%kcal)	-	
Gorduras (%kcal)	-	15%–30%
Gord. saturada (%kcal)	-	< 10%
Gord. poliinsaturadas (%kcal)	-	6%–10%
Gord. trans (%kcal)	-	< 1%
Sódio (mg)	-	≤ 2.300
Sódio / potássio	-	≤ 0,9
Cálcio (mg)	-	≥ 500
Frutas e hortaliças (g)	≥ 400	≥ 400
Alimentos ultraprocessados (g)	≤ observado	≤ observado

### Modelos de Programação Linear

Os modelos de otimização de dados por programação linear foram desenvolvidos para identificar a melhor solução (combinação de alimentos que irão compor as dietas otimizadas) que satisfaça ao conjunto de restrições de adequação nutricional ao menor custo possível. A função objetivo pode ser descrita segundo a seguinte formula


$$y=\sum_{i=1}^{i=g}\left(Q_{i}^{otm}\cdot {\text{preço}}_{i}\right)$$

onde: *g* é o número de alimentos, Q_*i*_^otm^é a quantidade do alimento *i* no cardápio otimizado e preço_*i*_é o preço do alimento por quilo.

### Análise Descritiva

Foram avaliadas as quantidades médias observadas e otimizadas dos seguintes grupos: feijão (feijão e leguminosas), laticínios (leite integral e desnatado, queijo, iogurte e outros laticínios), frutas, hortaliças, tubérculos (batata, mandioca e inhame); carne vermelha (carne bovina e suína), aves, peixes e frutos do mar, ovos, oleaginosas, óleos (manteiga e margarina); pães, bolos e biscoitos, arroz branco e integral, massas (macarrão, massa para lasanha e similares), farinhas (farinha de mandioca e farofa), molhos (molhos para salada e molhos para massas), bebidas adoçadas (refrigerantes, sucos industrializados e néctares), alimentos prontos para consumo (pizza, sanduíches e salgadinhos) e doces. A diferença relativa entre o custo otimizado e observado [(custo otimizado-custo observado)/custo observado × 100] foi avaliada segundo classes de renda para os dois períodos. Para permitir uma comparação direta entre os períodos, foi estimado o custo da dieta observada de 2008–2009 com os preços dos alimentos de 2017–2018. Todas as análises foram ponderadas pelos pesos amostrais, em que o peso do estrato correspondeu à somatória dos pesos de seus domicílios. Os modelos de otimização foram executados no software SAS.

## RESULTADOS

Os modelos só retornaram solução matemática viável para a maioria dos estratos após flexibilizar as quantidades máximas de frutas e hortaliças (itens individuais e grupos) em até três vezes o valor do percentil 95 da distribuição das médias de consumo. As restrições foram mantidas em percentis 5 e 95 para os demais alimentos e grupos.

O custo médio estimado com alimentação por pessoa/dia no Brasil em 2008–2009 foi de R$4,96, variando de R$3,80 a R$6,13 nos estratos de menor e maior renda. Em 2017–2018 foi de R$9,18, variando de R$7,53 a R$11,46 nos estratos de menor e maior renda. O custo otimizado médio – que considera somente modificações nas quantidades de frutas e hortaliças (modelo 1) – foi de R$4,62 em 2008–2009, variando de R$3,56 a R$5,56. Por sua vez, em 2017–2018 foi de R$8,69, variando de R$7,38 a R$10,42. Nesse modelo, a dieta otimizada custou em média 6.85% a menos que a dieta observada em 2008–2009 e 5,34% a menos que a dieta observada em 2017–2018. Quando incluídas restrições nutricionais para prevenção de doenças crônicas (modelo 2), o custo médio passou para R$ 5,08 em 2008–2009, variando de R$4,05 a R$6,09, e R$9,87 em 2017–2018, variando de R$8,65 a R$11,64. Neste modelo, a dieta otimizada custou em média 2,42% a mais que a dieta observada em 2008–2009 e 7,52% a mais que a dieta observada em 2017–2018. Os maiores incrementos no custo (em percentual) foram observados para o modelo 2 no período 2017–2018, especialmente nos estratos com rendimento *per capita* até um salário mínimo e na região nordeste. O custo da alimentação observada foi menor em ambos os períodos nas regiões Norte e Nordeste quando comparadas com as regiões Sul e Centro-Oeste ( Tabela 2 ).

**Tabela 2:** Custo médio (em Reais) das dietas observadas e otimizadas e variação percentual no custo da dieta otimizada segundo rendaa, região, restrições nutricionais (modelosb) e ano do inquéritoc.

	2008–2009			2017–2018		
	
	Observado^d^	Modelo 1^e^	Modelo 2^e^	Observado	Modelo 1	Modelo 2
Renda *per capita*						
< 0,5	3,80 (0,11)	3,56 (-6,31)	4,05 (+6,58)	7,53 (0,18)	7,38 (-1,96)	8,65 (+14,87)
0,5–1	4,42 (0,07)	4,15 (-6,10)	4,62 (+4,52)	8,31 (0,19)	8,02 (-3,49)	9,35 (+12,52)
1–2	4,91 (0,07)	4,66 (-5,09)	5,04 (+2,64)	9,13 (0,18)	8,67 (-5,04)	9,66 (+5,81)
> 2	6,13 (0,05)	5,56 (-9,29)	6,09 (-0,65)	11,46 (0,23)	10,42 (-9,08)	11,64 (+1,57)
Região						
Norte	5,28 (0,17)	4,91 (-7,00)	5,35 (+1,32)	8,68 (0,23)	8,10 (-6,68)	8,91 (+2,65)
Nordeste	4,37 (0,16)	3,96 (-9,38)	4,59 (+5,03)	8,40 (0,27)	7,82 (-6,90)	9,51 (+13,21)
Sudeste	5,18 (0,29)	4,91 (-5,21)	5,20 (+0,38)	9,26 (0,57)	8,88 (-4,10)	9,8 (+5,83)
Sul	5,22 (0,23)	4,81 (-7,85)	5,41 (+3,64)	10,32 (0,47)	9,62 (-6,78)	11,05 (+7,07)
Centro-Oeste	5,09 (0,2)	4,80 (-5,69)	5,27 (+3,53)	9,90 (0,39)	9,52 (-3,84)	10,47 (+5,76)
Brasil	4,96 (0,16)	4,62 (-6,85)	5,08 (+2,42)	9,18 (0,28)	8,69 (-5,34)	9,87 (+7,52)

^a^Renda domiciliar *per capita* em salários mínimos.

^b^Modelo 1: adequação de frutas e hortaliças. Modelo 2: adequação de frutas e hortaliças, macronutrientes e gorduras, cálcio, sódio e potássio. Quantidade de alimentos ultraprocessados menor ou igual à quantidade observadas para ambos os modelos.

^c^n = 108 estratos para ambos os períodos.

^d^Média (erro-padrão)

^e^Custo em Reais (percentual em relação ao custo observado no período) calculado como: [(custo otimizado-custo observado)/custo observado × 100.

Na menor faixa de renda, o custo de dieta otimizada (modelo 1) em relação ao custo observado variou entre os estratos de -21% a +6% em 2008–2009 e -15% a +11% em 2017–2018. Para o modelo 2, essa variação foi de -11% a +25% em 2008–2009 e -11% a +34% em 2017–2018. Ainda nesse estrato de renda o custo médio da alimentação saudável em 2008-2009 foi maior que o custo da alimentação observada no período em 8% dos estratos do modelo 1 e em 71% dos estratos do modelo 2. Em 2017–2018 esses valores foram 16% no modelo 1 e 82% no modelo 2. O incremento no custo referente à dieta otimizada alcançou 34% em relação ao custo observado nos estratos de até 1 salário mínimo *per capita* no modelo 2 ( Figura 1 ).

**Figura 1:** Diferença relativa (%)a entre os custos otimizados e observados nos estratos segundo rendab, restrições nutricionais (modelosc) e ano do inquérito.

^a^Calculada como [(custo otimizado-custo observado)/custo observado] × 100.

^b^Renda domiciliar *per capita* em salários mínimos.

^c^Modelo 1: adequação de frutas e hortaliças. Modelo 2: adequação de frutas e hortaliças, macronutrientes e gorduras, cálcio, sódio e potássio. Quantidade de alimentos ultraprocessados menor ou igual à quantidade observadas para ambos os modelos.

Se a dieta de 2017–2018 fosse a mesma praticada em 2008–2009, isto é, dieta de 2008–2009 com os preços de 2017–2018, seu custo seria de R$9,61, portanto, R$ 0,43 mais cara que o estimado em 2017–2018.

No primeiro inquérito, o consumo de arroz, laticínios, feijão, doces, peixes e bebidas adoçadas foi maior que no segundo inquérito. De uma forma geral, as modificações na dieta para acomodar o aumento nas quantidades de frutas e hortaliças foram semelhantes entre os dois períodos: redução nos laticínios, frango, molhos, ovos, carne vermelha, *snacks* e bebidas adoçadas; e aumento no arroz e farinhas. Para acomodar também as recomendações para prevenção de DCNT, foi necessário aumento mais expressivo de frutas e hortaliças, totalizando 535g em 2008–2009 e 486g em 2017–2018, além de aumento nos tubérculos, feijão, laticínios e redução em margarinas e manteigas, massas, pães, biscoito e bolo ( Tabela 3 ).

**Tabela 3:** Média de consumo em gramas de alimentos e grupos de alimentos nas dietas observadas e otimizadas segundo restrições nutricionais (modelosa) e ano de inquéritob.

	2008–2009			2017–2018		
	
	Observado^c^	Modelo 1	Modelo 2	Observado^c^	Modelo 1	Modelo 2
Arroz	180,80 (5,86)	194,21	180,14	149,54 (4,5)	160,77	148,72
Biscoitos	16,54 (0,75)	14,33	9,71	15,49 (0,73)	13,76	10,11
Bolos	12,96 (0,69)	13,40	14,99	11,19 (0,55)	8,84	8,39
Laticínios	134,35 (5,34)	112,11	147,06	97,65 (4,64)	79,35	117,41
Doces	21,51 (1,09)	14,48	16,48	14,5 (0,94)	7,43	8,82
Farinhas	9,18 (1,89)	14,95	15,82	7,83 (1,59)	11,97	12,31
Feijões	203,55 (8,14)	203,23	217,03	175,19 (6,47)	179,18	189,79
Aves	36,15 (1,32)	29,38	35,04	50,57 (1,76)	44,29	53,64
Frutas	177,51 (6,96)	259,75	340,64	166,25 (5,83)	251,64	301,02
Hortaliças	60,32 (3,31)	140,25	194,57	66,8 (3,19)	148,36	185,89
Massas	43,89 (2,13)	46,59	33,51	40,64 (1,69)	43,76	31,76
Molhos	4,46 (0,59)	1,45	1,41	4,52 (0,35)	1,40	2,07
Oleaginosas	0,18 (0,03)	0,31	0,34	0,34 (0,06)	0,52	0,58
Óleos	6,32 (0,19)	8,52	3,52	8,26 (0,31)	8,28	4,79
Ovos	12,35 (0,72)	8,84	13,11	12,74 (0,45)	8,76	12,37
Pães	56,76 (1,49)	58,80	42,21	57,94 (1,44)	60,17	49,53
Peixes	28,34 (3,75)	21,49	26,60	18,78 (2,36)	12,47	20,99
Carne vermelha	95,65 (2,43)	78,04	73,81	99,02 (2,27)	82,92	85,83
Prontos para consumo	20 (1,58)	14,98	11,54	22,16 (1,25)	15,97	13,94
Beb. Adoçadas	123,23 (9,62)	116,53	111,07	80,52 (3,43)	74,17	69,90
Tubérculos	30,61 (1,73)	27,15	42,64	33,7 (1,79)	29,41	42,65

^a^Modelo 1: adequação de frutas e hortaliças. Modelo 2: adequação de frutas e hortaliças, macronutrientes e gorduras, cálcio, sódio e potássio. Quantidade de alimentos ultraprocessados menor ou igual à quantidade observadas para ambos os modelos.

^b^n = 108 estratos para ambos os períodos.

^c^Média (erro-padrão).

O impacto do aumento de frutas e hortaliças nas quantidades de nutrientes foi mais evidente para fibra, vitamina A, vitamina C e folato, que aumentaram em pelo menos 10% em relação à ingestão observada para os dois períodos. Por outro lado, houve redução em ambos os períodos. Ao incluir as restrições nutricionais do modelo 2, houve aumento mais expressivo para esses e outros nutrientes, como vitamina E, cobre, zinco e magnésio, além do aumento no cálcio e potássio e redução no sódio, nutrientes que tiveram as quantidades definidas no modelo 2 ( Tabela 4 ).

**Tabela 4:** Média de ingestão de energia e nutrientes nas dietas observadas e otimizadas segundo restrições nutricionais (modelosa) e ano de inquéritob.

	2008–2009			2017–2018		
	
	Observado^c^	Modelo 1	Modelo 2	Observado^c^	Modelo 1	Modelo 2
Energia (kcal)	1.696,7 (13,4)	1.696,8	1.696,8	1.699,9 (10,5)	1.699,9	1.699,9
Carboidratos (g)	2.32,5 (1,65)	252,5	253,6	230,1 (2,0)	251,3	244,2
Proteínas (g)	77,5 (0,8)	69,8	73,2	78,5 (0,6)	71,2	79,4
Gorduras (g)	56,3 (0,7)	52,0	50,8	56,8 (0,6)	51,8	51,8
Gord. Monoinsaturadas (g)	17,2 (0,2)	15,5	15,3	18,4 (0,3)	17,2	15,8
Gord. Poliinsaturadas (g)	13,4 (0,2)	12,5	12,8	14,2 (0,2)	13,0	13,1
Gord. Saturadas (g)	19,3 (0,3)	17,9	16,8	17,8 (0,2)	15,5	16,3
Gord. Trans (g)	1,43 (0,03)	1,37	1,09	1,33 (0,02)	1,2	1,07
Fibras (g)	25,1 (0,4)	29,1	32,5	22,7 (0,3)	26,9	29,5
Cálcio (mg)	445,1 (12,7)	401,9	508,5	394,1 (12,6)	353,4	500,8
Cobre (mg)	1,45 (0,03)	1,55	1,70	1,29 (0,02)	1,40	1,54
Ferro (mg)	11,3 (0,1)	11,3	11,1	10,8 (0,1)	10,8	11,0
Fósforo (mg)	1.001,5 (11,1)	920,8	1.011,8	972,6 (6,8)	895,4	1035
Magnésio (mg)	258,7 (3,4)	272,5	300,2	239,7 (2,0)	254,6	282,2
Potássio (mg)	2.118,3 (28,8)	2.296,7	2.761,3	2.011,0 (19,2)	2.196,6	2.616,9
Sódio (mg)	2.505,6 (29,1)	2.382	2.238,4	2.376,6 (21,2)	2.264,9	2.260,5
Zinco (mg)	10,8 (0,1)	10.1	10,2	10,8 (0,1)	10,2	10,7
Niacina (mg)	13,8 (0,3)	12.3	12,8	15,1 (0,2)	13,4	15,1
Folato (mcg)^d^	446,5 (6,7)	477,7	491,5	407,4 (5,3)	439,3	464,2
Vitamina A (EAR)^e^	518,8 (26,4)	603,1	732,2	429,8 (17,7)	475,3	639,5
Vitamina B1 (mg)	0,89 (0,01)	0,89	0,89	0,93 (0,01)	0,91	0,96
Vitamina B12 (mg)	5,3 (0,18)	4,42	4,64	4,16 (0,12)	3,42	4,02
Vitamina B2 (mg)	1,08 (0,02)	1,03	1,06	0,93 (0,01)	0,89	0,96
Vitamina B6 (mg)	0,66 (0,01)	0,7	0,65	0,62 (0,01)	0,66	0,65
Vitamina C (mg)	124,9 (3,7)	243,4	339,5	117,4 (3,5)	233,3	270,3
Vitamina D	2,66 (0,07)	2,31	2,77	2,11 (0,04)	1,8	2,33
Vitamina E (mg)	5,78 (0,07)	6,34	7,09	6,11 (0,08)	6,76	6,81

^a^Modelo 1: adequação de frutas e hortaliças. Modelo 2: adequação de frutas e hortaliças, macronutrientes e gorduras, cálcio, sódio e potássio. Quantidade de alimentos ultraprocessados menor ou igual à quantidade observadas para ambos os modelos.

^b^n = 108 estratos para ambos os períodos.

^c^Média (erro-padrão).

^d^Em equivalentes dietéticos de folato (DFE).

^e^Em equivalentes de atividade de retinol.

## DISCUSSÃO

Neste estudo foi estimado o menor custo de uma alimentação saudável e culturalmente aceitável para os períodos de 2008–2009 e 2017–2018 considerando as variações nas preferências alimentares e nos preços dos alimentos no país. A adequação de frutas e hortaliças pôde ser alcançada com uma redução de 6,8% e 5,3% em relação ao custo observado com alimentação em 2008–2009 e 2017–2018, respectivamente. A inclusão de restrições relativas à prevenção de DCNT resultou em aumento mais expressivo de 7,5% em 2017–2018. Essas diferenças no impacto no custo da adoção de uma alimentação saudável entre os períodos podem ser explicadas por uma variação desigual dos preços entre os grupos de alimentos. A partir do início dos anos 2000 em diante o preço dos alimentos ultraprocessados vem decrescendo em relação ao preço dos alimentos *in natura* e minimamente processados^13^. Além disso, as médias de consumo de alguns alimentos marcadores de alimentação saudável, como laticínios, frutas, feijão e peixes, foram um pouco maiores em 2008–2009. Somente a título de comparação, o custo da dieta de 2008–2009, se adquirida com preços relativos a 2017–2018, seria R$9,61, isto é, 4,7% mais cara que o custo da dieta estimado de 2017–2018, que foi R$9,18. Assim, a adequação da dieta demandou menores modificações e, consequentemente, menores custos, particularmente no modelo 2 em que a composição nutricional do alimento também foi considerada na otimização.

A redução no custo médio da alimentação com quantidade adequada de frutas e hortaliças presentes no modelo 1 foi possível pela redução na quantidade de outros alimentos. O modelo otimiza as quantidades dos alimentos dentro da menor variação possível para incluir a quantidade definida de frutas e hortaliças, ou seja, a adequação não aumenta o custo contanto que outras modificações na alimentação também sejam feitas. Ainda que na média do país e na média de cada faixa de renda a adequação da quantidade de frutas e hortaliças não tenha resultado em aumento no custo da dieta, em vários estratos o incremento no custo foi necessário. Isso indica uma variação importante no impacto da adequação da dieta no custo pelo país. Particularmente no último inquérito e especialmente nas faixas de menor renda, o incremento no custo alcançou foi maior que 30% em relação ao custo observado para alguns estratos. De fato, adequar a quantidade de frutas e hortaliças na dieta das famílias de baixa renda parece não ser possível sem aumento no custo com alimentação. Estudo similar utilizando dados do inquérito de 2008–2009 com objetivo de reduzir o percentual da participação calórica de AUP e adequar ao máximo possível a quantidade de frutas e hortaliças em 400g/dia sem aumento no custo da dieta, atingiu 350g nos estratos de até meio salário mínimo *per capita* . Nos estratos de maior renda, essa quantidade chegou a 700g^14^. Para o modelo 2, o incremento ocorreu em todos os estratos de renda, exceto entre os de maior renda em 2008–2009. A implicação imediata destes resultados é que, para um percentual considerável de famílias, melhorar a alimentação resulta em maior gasto com alimentação.

O custo mínimo com alimentação adequada foi obtido considerando limites mínimos e máximos, denominados aqui restrições de aceitabilidade. Essas restrições delimitam o quanto cada alimento pode “desviar” da quantidade relatada em cada um dos períodos. Não havendo informações sobre preferências alimentares na população, os limites de aceitabilidade foram derivados da distribuição de consumo na própria população. O racional é que, caso as pessoas tenham relatado esses itens, isso significa que eles são aceitáveis na população. Entretanto, os limites definidos a partir das distribuições (neste caso, percentil 5 e 95) foram escolhidos arbitrariamente, consistindo, portanto, em uma limitação em estudos que envolvem aspectos socioculturais da dieta – ainda que seja um procedimento comum em estudos similares a este^6 , 15^. Além da dimensão sociocultural, a definição do que é uma alimentação saudável também tem importante impacto nos custos. Quando a adequação de vitaminas e minerais é incluída entre as restrições dos modelos, o custo é em média 20% maior em relação ao gasto observado, ocorrendo em todos os níveis de renda^5^. No entanto, o custo de uma alimentação adequada em micronutrientes reduziram na medida em que restrições de aceitabilidade mais flexíveis foram sendo introduzidas aos modelos. Ou seja, quanto maior a tolerância à modificação no consumo, menor o custo final. Destaca-se, no entanto, que para as famílias de menor renda, o custo otimizado foi sempre maior que o observado para todos os limites de aceitabilidade impostos, o que não ocorreu nos demais estratos de renda^5^. Assim, deve-se ter em mente que o custo estimado neste estudo corresponde ao menor custo relacionado ao menor desvio em relação à dieta habitual em cada um dos períodos. Tal como nos demais estudos, o custo se reduz à medida que modificações mais drásticas são toleradas.

No presente estudo foram utilizados dois conjuntos de restrições nutricionais. O primeiro diz respeito somente às quantidades de alimentos, caracterizando-se como um cenário mais realista, uma vez que é mais provável que as pessoas façam as compras observando as quantidades de cada alimento na cesta. Por outro lado, o cenário do modelo 2 é importante para avaliar o quanto a adesão a demais recomendações para prevenção de DCNT demandaria de modificações na alimentação e no próprio orçamento. Trata-se de uma informação importante para profissionais de saúde e para elaboração de guias que devem considerar estratégias para alcance de objetivos de ingestão de determinados componentes da dieta ao menor custo e maior aceitabilidade.

Algumas questões metodológicas devem ser consideradas na interpretação desses resultados. Assumimos que os preços dos alimentos como bolos, sanduiches, doces, pizzas, lanches e salgados fritos e assados correspondem ao preço do alimento adquirido pronto para consumo, ainda que possam ser itens preparados em casa a partir da compra dos ingredientes. Nesse caso, o preço médio desses itens estará superestimado à medida que estas preparações são feitas em casa. Por outro lado, para preparações à base de cereais, leguminosas, carnes e massas, como arroz, feijão, lasanha etc., foi assumido a somatório do preço de seus ingredientes, ainda que possam ter sido adquiridos prontos para consumo em mercados ou restaurantes. Nesse caso, o preço médio pode estar subestimado, pois espera-se que preparações custem mais quando adquiridas prontas do que preparadas em casa. O sub-relato do consumo – comum em inquéritos alimentares – certamente leva a uma subestimativa do custo da alimentação observada^16^. No entanto, tanto o custo observado quanto o otimizado foram calculados para a mesma quantidade média de calorias em cada período, sendo, portanto, impactados da mesma forma pelo sub-relato. Assim, na ausência de sub-relato, os custos observados e esperados seriam maiores que os relatados aqui, não sendo, contudo, possível afirmar que a diferença entre eles permaneceria na mesma proporção que o encontrado no estudo. O sub-relato pode ocorrer de forma diferente entre os alimentos^17^. Caso tenha sido maior nas frutas e hortaliças, o incremento no custo pelo aumento na quantidade dos alimentos deste grupo seria menor que o obtido aqui. Cabe também destacar que as mudanças nas dietas otimizadas acarretam não somente em mudanças econômicas e culturais da alimentação, mas também em aspectos de conveniência. O aumento na ingestão de frutas e vegetais implica visitas mais frequentes ao mercado, além de maior tempo gasto no preparo e cocção. Variáveis não consideradas neste estudo.

Um dos pontos principais deste estudo foi considerar a grande variação de preços e de padrões de consumo pelo país, otimizando o custo da dieta adequada mais semelhante possível à dieta observada em cada estrato e em cada período. A avaliação apenas da média do país omite em que extensão a adequação da dieta impacta os estratos nas diferentes regiões do país. Entretanto, em função da grande variação no dia-a-dia no consumo alimentar, foi necessário agrupar os indivíduos em estratos, cuja média do consumo de um dia é uma boa aproximação da média de consumo usual no grupo^18^.

Concluindo, este estudo indica que a adequação da quantidade de frutas e hortaliças na dieta pode não acarretar aumento de custo médio tanto em 2008–2009 quanto em 2017–2018. No entanto, para um percentual significativo dos estratos avaliados, a adequação demandaria aumento no gasto com alimentos, particularmente no período de 2017–2018. Quando a adequação de cálcio, sódio e potássio também são introduzidas aos modelos de otimização, observou-se um aumento no custo da dieta nos dois períodos examinados.
